# Reasons for not receiving the HPV vaccine among eligible adults: Lack of knowledge and of provider recommendations contribute more than safety and insurance concerns

**DOI:** 10.1002/cam4.3192

**Published:** 2020-06-01

**Authors:** Joël Fokom Domgue, Sonia A. Cunningham, Robert K. Yu, Sanjay Shete

**Affiliations:** ^1^ Department of Epidemiology The University of Texas MD Anderson Cancer Center Houston TX USA; ^2^ Division of Cancer Prevention and Population Science The University of Texas MD Anderson Cancer Center Houston TX USA; ^3^ Department of Gynecologic Oncology and Reproductive Medicine The University of Texas MD Anderson Cancer Center Houston TX USA; ^4^ Department of Biostatistics The University of Texas MD Anderson Cancer Center Houston TX USA

**Keywords:** adult population, HPV vaccination, necessity, reasons, safety, Texas

## Abstract

**Background:**

The upward trends of vaccine exemptions in Texas are alarming. While HPV vaccine rates in this State are among the lowest nationwide, factors that contribute to the low HPV vaccination uptake among adults remain unknown. In this study, we examined the main reasons for not receiving HPV vaccination among age‐eligible adults.

**Methods:**

The Texas health screening survey (2018), a multistage area probability design‐based survey of a representative sample of Texas residents, was used to identify 907 eligible adults (age ≥ 18 years) respondents, including 724 women aged ≤ 26 years in 2007 (≤38 years in 2018), and 183 men aged ≤ 21 years in 2011 (≤28 years in 2018). Participants who reported having never received an HPV shot, where asked the main reason for not receiving the vaccine.

**Results:**

Overall, 58.5% (95%CI: 55.1‐62.0) of vaccine eligible adults reported having never received the HPV vaccine. The most commonly reported reasons for not receiving it were: did not know about the vaccine (18.5% (14.9‐22.1)), and provider did not recommend (14.1% (10.9‐17.4)). In contrast, commonly perceived reasons such as: safety concerns (7.2% (4.8‐9.5)), lack of insurance (3.4% (1.7‐5.1), and concerns about increasing sexual activity if vaccinated (0.2% (0.0‐0.5)), were less frequently reported.

**Conclusion:**

Among vaccine‐eligible adults, safety and sexuality concerns do not appear to be the prime factors underlying low HPV vaccination rates. Rather than emphasizing them, educational interventions should aim at improving vaccine's knowledge, and enhancing provider recommendations on the necessity of HPV vaccination.

## Graphical abstract

Among HPV‐vaccine eligible adults, lack of knowledge about the vaccine and lack of provider recommendations are the most commonly reported reasons for not receiving the HPV vaccine. In this population, educational interventions should focus on enhancing HPV vaccine's knowledge and provider recommendations of the necessity and awareness of HPV vaccination.

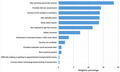

## 1 INTRODUCTION

In the United States (US), the human papillomavirus (HPV) causes about 33 700 cancers each year, including cancers of the cervix, vagina, vulva, oropharynx, and anus.^1^ Following approval by the Food and Drug Administration (FDA), the American Committee on Immunization Practices (ACIP) recommended HPV vaccination for women in 2007 and for men in 2011.^2^ Despite constant efforts to promote knowledge of and accessibility to HPV vaccine, uptake in the US remains suboptimal.

Parental approval plays a key role in receipt of HPV vaccines by minor children.^3^ When children become legally adults (≥18 years of age), however, they no longer need approval from their parents/guardians to get vaccinated. Until 2018, HPV vaccination was recommended for US adults who have not previously been adequately immunized, through age 21 for men and 26 for women.^2^, ^4^ Despite these guidelines, HPV vaccination coverage in eligible adults remains low in the US. In 2016, only 8.6% of women and 2.7% of men aged 19‐26 years who had not received HPV vaccination prior to age 19, reported having had at least one dose of HPV vaccine.^5^ This is a matter of concern, as 64% to 82% of incident HPV DNA detected in mid‐age US adults (25 to 60 years of age) who engage in new sexual partnerships is attributable to newly acquired infection.^6^, ^7^

There is a strong body of evidence suggesting that HPV vaccine is safe and effective in preventing HPV infection and HPV‐related disease in both teenagers and older age groups.^8^, ^9^, ^10^ Based on these data, the FDA approved the expanded use of HPV vaccine in 2018 to include individuals 27 through 45 years.^11^ Considering the increasing incidence of HPV‐associated cancers in the US, particularly oropharyngeal and anal cancers for which population‐based screening is not endorsed,^12^ the ACIP recently recommended HPV vaccination in this age group as part of the shared decision‐making process between patients and clinicians.^13^ This provides additional opportunities to people who did not receive the vaccine as minors, to benefit from it later in their life. In contrast to the compelling literature examining parents’ reasons for not consenting their children to receive HPV vaccination,^14^ little is known about the reasons why eligible, independent adults still do not get the HPV vaccine.

In the state of Texas, the second most populated region of the US, the proportion of parents/guardians who exempt their children from vaccination for nonmedical reasons has risen over the last decade,^15^ to the point where public health officials anticipate resurging outbreaks of certain preventable diseases among vulnerable populations.^16^, ^17^ With only 43.5% of adolescents were being up‐to‐date on their HPV vaccine series in 2018, Texas is lagging behind most US states on HPV vaccination.^18^ The rates are even lower among vaccine‐eligible adults.^8^ However, factors that contribute to the suboptimal HPV vaccine uptake in this group have not been previously assessed from an individual's perspective. In this study, we examined: (i) the proportion and characteristics of vaccine‐eligible adults in Texas who have never received HPV vaccine; and (ii) their reasons for not getting vaccinated. Understanding these motivations may help identify effective interventions to improve HPV vaccine coverage in this population.

## 2 METHODS

### 2.1 Study population and recruitment procedure

Our study population was selected from a representative sample of the Texas population; the enrolment procedure has been described in detail elsewhere.^19^, ^20^ Briefly, a nonprobability sample of 2050 respondents to the Texas health screening survey was collected between 5 February and 5 March 2018, using strata set to mirror Texas demographics for sex, ethnicity, race, and income. However, oversampling of non‐Hispanic Blacks (NHBs) was conducted to ensure more accurate estimation in this minority group. The non‐Hispanic White (NHW) category consisted of those selecting White as the sole race and the NHB category as those selecting Black/African American (either alone or in addition to other races). The recruitment target included 60% urban and 40% rural respondents, categorized by matching ZIP code to county designations.^21^, ^22^ The instrument was prepared in English with Mexican Spanish translation with the help of Masterword Services, Inc, Houston, TX, and loaded into the Qualtrics online survey platform. Qualtrics managed hosting and compensation of the survey using opt‐in panelists. The study protocol (PA16‐0724) was approved by MD Anderson's Institutional Review Board. The present study focused on the population of adult respondents who were eligible for HPV vaccination, that is, women participants who were aged ≤ 26 years, and men participants who were aged ≤ 21 years when HPV vaccination was first recommended in the US (ie in 2007 for women and 2011 for men). Thus, our study sample consisted of 907 respondents, including 724 women aged ≤ 26 years in 2007 (≤38 years at the time of survey), and 183 men aged ≤ 21 years in 2011 (≤28 years at the time of survey).

### 2.2 Outcome measures

The main outcomes for this study were: (a) self‐reported receipt of HPV vaccine, and (b) self‐reported reasons for not receiving HPV vaccine. To measure these outcomes, we adapted two validated questions from the National Immunization Survey‐Teen (NIS‐Teen). In the Texas health screening survey, these questions were directed to adult respondents who were or have been eligible to receive HPV vaccination. We first asked: (a) *HPV is a common sexually transmitted virus that can cause genital warts and cervical and other types of cancer in men and women. Vaccines to prevent some HPV infections are available for men and women 9 ‐ 26 years of age and are sometimes called the HPV shot, Cervarix, or Gardasil. Have you ever received the HPV shot, Cervarix, or Gardasil?* The possible responses were: *Yes/No/Don't know*. Participants who responded “No” were asked: (b) *What is the main reason you have not received the HPV shot/ vaccine?* Possible responses were: *Provider did not recommend (a), Did not know about the vaccine (b), Vaccine not needed or necessary (c), Not required to get the vaccine (d), Safety concerns (e), Provider indicated could vaccinate later (f), Uninsured or insurance doesn't fully cover shots, or co‐pay or other costs too high (g), Shot could be painful (h), Vaccine not available (i), Difficulty making or getting to appointments/ transportation issues (j), Concern about increasing sexual activity if receive shot (k), Not sexually active (l), Some other reason (m)*.

### 2.3 Covariates

Covariates were selected based on their potential influence on HPV vaccination uptake, including: socio‐demographic factors of age, ethnicity/race, nativity, educational attainment, marital status, occupation, rurality, and household income; behavioral and mental factors; and health‐related variables such as smoking status, health coverage, vaccination against Hepatitis B virus (HBV), hormonal contraception use, and family history of any cancer.

### 2.4 Statistical analysis

Data were weighted by ICF International, Inc, Fairfax, Virginia using a three‐dimensional raking approach with iterative post‐stratification based on sex, age, and race/ethnicity.^23^ We used means and standard deviation (continuous variables), weighted percentages and weighted 95% confidence interval (categorical variables), to describe the study population by HPV vaccination status. The reasons for having never received HPV vaccine were described according to the socio‐demographic, health‐related, and behavioral characteristics of the study population.

## 3 RESULTS

### 3.1 Characteristics of the study population

Table 1 describes the sociodemographic, health‐related, and behavioral characteristics of the study sample, stratified by HPV vaccine status. Of the 907 adult respondents included in this study, 198 [weighted percentage: 21.6% (weighted 95% confidence interval: 18.7‐24.5%)] reported having ever received (at least one dose of) HPV vaccination, while 530 [58.5% (55.1‐62.0%)] reported having never received this vaccine. The remaining 177 respondents [19.7% (16.8‐22.5%)] reported not knowing if they have received the HPV vaccine.

**TABLE 1:** Characteristics of the Study Population according to HPV vaccination status

Have you ever received the HPV shot, Cervarix, or Gardasil?								
	Yes		No		Don't know		Total	
Variables	N	% (95% CI)	N	% (95% CI)	N	% (95% CI)	N^a^	% (95% CI)
Age (y)								
18‐28	151	78.9 (73.1‐84.7)	310	63.8 (59.5‐68.1)	131	79.4 (73.5‐85.3)	594	70.2 (67.1‐73.3)
29‐38	47	21.1 (15.3‐26.9)	220	36.2 (31.9‐40.5)	46	20.6 (14.7‐26.5)	313	29.8 (26.7‐32.9)
Sex								
Female	165	73.3 (65.6‐80.9)	417	68.5 (63.8‐73.3)	140	65.7 (57.3‐74.2)	724	69.1 (65.4‐72.7)
Male	33	26.7 (19.1‐34.4)	113	31.5 (26.7‐36.2)	37	34.3 (25.8‐42.7)	183	30.9 (27.3‐34.6)
Ethnicity/ Race								
Black, non‐Hispanic	41	10.1 (6.9‐13.4)	133	12.9 (10.6‐15.3)	39	10.4 (7.0‐13.9)	213	11.8 (10.1‐13.5)
Hispanic	108	56.4 (48.9‐63.9)	230	46.7 (42.1‐51.3)	99	59.4 (51.4‐67.4)	439	51.4 (47.9‐54.9)
Others	11	6.4 (2.6‐10.3)	45	8.8 (6.3‐11.4)	11	6.2 (2.5‐10.0)	67	7.8 (5.9‐9.7)
White, non‐Hispanic	38	27.0 (19.7‐34.3)	122	31.5 (27.0‐36.1)	28	23.9 (16.2‐31.6)	188	29.0 (25.5‐32.5)
Born In USA								
No	16	7.4 (3.8‐11.1)	44	7.5 (5.2‐9.7)	22	12.0 (7.0‐17.0)	82	8.3 (6.5‐10.2)
Yes	182	92.6 (88.9‐96.2)	486	92.5 (90.3‐94.8)	155	88.0 (83.0‐93.0)	825	91.7 (89.8‐93.5)
Education								
No greater than 12 y or completed high school	52	27.7 (20.9‐34.4)	181	35.1 (30.6‐39.5)	88	51.6 (43.6‐59.7)	322	36.8 (33.3‐40.2)
Post high school training or some college	69	34.5 (27.3‐41.7)	191	35.1 (30.7‐39.5)	53	30.3 (22.7‐37.8)	313	33.9 (30.6‐37.3)
College/Postgraduate	77	37.8 (30.5‐45.1)	157	29.9 (25.6‐34.1)	36	18.1 (12.1‐24.0)	271	29.3 (26.1‐32.5)
Marital status								
Single/Widowed/ Divorced/Separated	119	61.0 (53.7‐68.3)	294	56.8 (52.2‐61.4)	109	61.8 (53.9‐69.6)	524	58.8 (55.3‐62.3)
Living as Married/Married	76	39.0 (31.7‐46.3)	227	43.2 (38.6‐47.8)	66	38.2 (30.4‐46.1)	369	41.2 (37.7‐44.7)
Occupation								
Employed	122	59.7 (52.2‐67.1)	299	57.2 (52.7‐61.8)	73	40.4 (32.5‐48.4)	494	54.3 (50.8‐57.9)
Homemaker/Unemployed/Disabled	48	24.0 (17.7‐30.2)	151	28.3 (24.2‐32.4)	69	40.2 (32.2‐48.1)	269	29.7 (26.5‐32.9)
Student/Retired/Other	28	16.3 (10.3‐22.4)	78	14.5 (11.2‐17.8)	35	19.4 (13.1‐25.8)	142	15.9 (13.3‐18.6)
Income								
≤$19 999	46	24.1 (17.5‐30.6)	158	28.7 (24.6‐32.9)	73	40.6 (32.7‐48.5)	278	30.1 (26.9‐33.3)
$20 000 to $49 999	70	33.8 (26.8‐40.9)	169	31.3 (27.0‐35.5)	60	34.6 (26.8‐42.4)	299	32.4 (29.1‐35.7)
$50 000 to $74 999	36	18.3 (12.5‐24.1)	101	17.9 (14.5‐21.3)	21	11.4 (6.5‐16.2)	159	16.8 (14.2‐19.3)
≥$75 000	46	23.8 (17.3‐30.3)	102	22.1 (18.1‐26.2)	23	13.4 (7.8‐19.0)	171	20.7 (17.7‐23.7)
Residence								
Rural	59	34.9 (27.5‐42.4)	184	39.8 (35.2‐44.4)	69	43.1 (35.1‐51.2)	313	39.4 (35.9‐43.0)
Urban	139	65.1 (57.6‐72.5)	346	60.2 (55.6‐64.8)	108	56.9 (48.8‐64.9)	594	60.6 (57.0‐64.1)
Hormonal contraception^b^								
No	40	16.2 (11.2‐21.3)	161	25.3 (21.6‐29.0)	50	23.7 (17.5‐29.9)	252	23.1 (20.4‐25.8)
Yes	125	57.1 (49.4‐64.8)	256	43.2 (38.7‐47.7)	90	42.0 (34.3‐49.7)	471	45.9 (42.4‐49.4)
Smoking								
Current smokers	43	22.4 (16.1‐28.8)	111	22.7 (18.7‐26.7)	41	25.0 (17.9‐32.1)	195	23.1 (20.0‐26.1)
Former smokers	16	8.9 (4.6‐13.3)	39	7.3 (5.0‐9.7)	13	9.2 (4.2‐14.2)	68	8.0 (6.1‐10.0)
Never smokers	139	68.6 (61.6‐75.7)	380	70.0 (65.6‐74.3)	123	65.8 (58.0‐73.6)	643	68.9 (65.5‐72.2)
Health care coverage								
No	58	31.5 (24.4‐38.7)	201	37.3 (32.8‐41.8)	84	47.0 (38.9‐55.1)	343	38.0 (34.5‐41.4)
Yes	139	68.5 (61.3‐75.6)	329	62.7 (58.2‐67.2)	93	53.0 (44.9‐61.1)	561	62.0 (58.6‐65.5)
Hepatitis B Virus vaccination								
No	53	26.3 (19.6‐32.9)	350	66.9 (62.6‐71.3)	77	43.5 (35.4‐51.5)	480	53.4 (49.9‐56.9)
Yes	145	73.7 (67.1‐80.4)	180	33.1 (28.7‐37.4)	100	56.5 (48.5‐64.6)	425	46.4 (42.9‐49.9)
Family History of any cancer								
No	48	22.2 (16.0‐28.3)	206	36.5 (32.1‐41.0)	53	28.1 (21.1‐35.1)	307	31.8 (28.5‐35.0)
Not sure	19	11.0 (5.8‐16.2)	57	12.7 (9.4‐16.0)	29	17.3 (11.0‐23.5)	105	13.2 (10.7‐15.8)
Yes	131	66.9 (59.7‐74.0)	266	50.8 (46.1‐55.4)	95	54.6 (46.6‐62.6)	492	55.0 (51.5‐58.5)

^a^ 2 participants did not provide information about their vaccination status.

^b^ Male participants did not respond to the question about hormonal contraception use.

HPV unvaccinated vs vaccinated respondents were more frequently: aged between 29 and 38 years [36.2% (31.9‐40.5) vs 21.1% (15.3‐26.9)]; males [31.5% (26.7‐36.2) vs [26.7% (19.1‐34.4)], and NHWs [31.5% (27.0‐36.1) vs 27.0% (19.7‐34.3)]. HPV unvaccinated respondents were also more likely to: have a level of education not greater than 12 years [35.1% (30.6‐39.5) vs 27.7% (20.9‐34.4)]; and to reside in rural areas [(39.8% (35.2‐44.4) vs 34.9% (27.5‐42.4)], compared with their vaccinated counterparts. (Table 1).

HPV unvaccinated respondents were less likely to: use hormonal contraception [43.2% (38.7‐47.7) vs 57.1% (49.4‐64.8)]; have health insurance [62.7% (58.2‐67.2) vs 68.5% (61.3‐75.6)]; be vaccinated against HBV [33.1% (28.7‐37.4) vs 73.7% (67.1‐80.4)]; and have a family history of cancer [50.8% (46.1‐55.4) vs 66.9% (59.7‐74.0)], compared with HPV vaccinated respondents. (Table 1).

### 3.2 Reasons for having not received HPV vaccination

**FIGURE 1:**
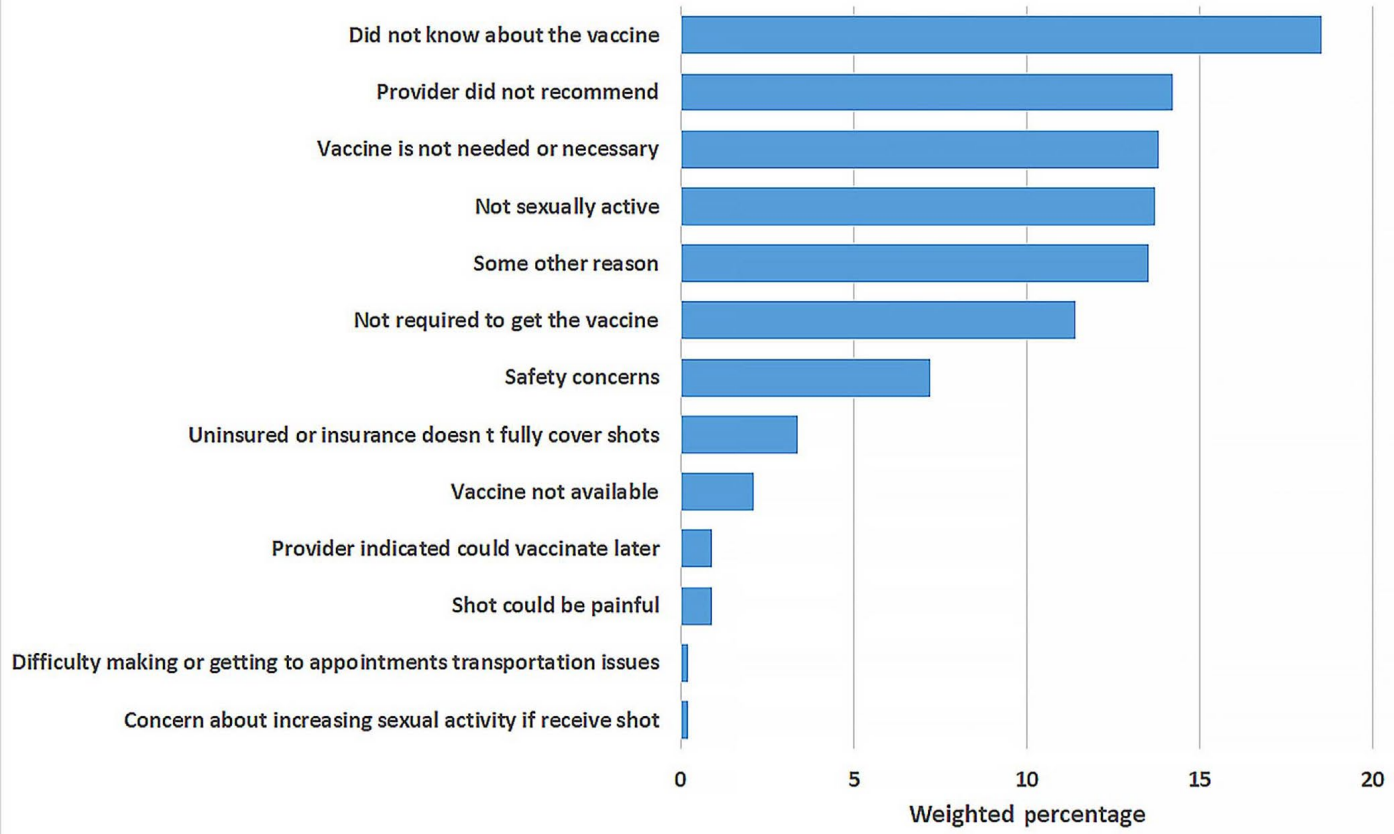
Main Reasons for not getting the HPV vaccine among vaccine‐eligible adults, overall

**FIGURE 2:**
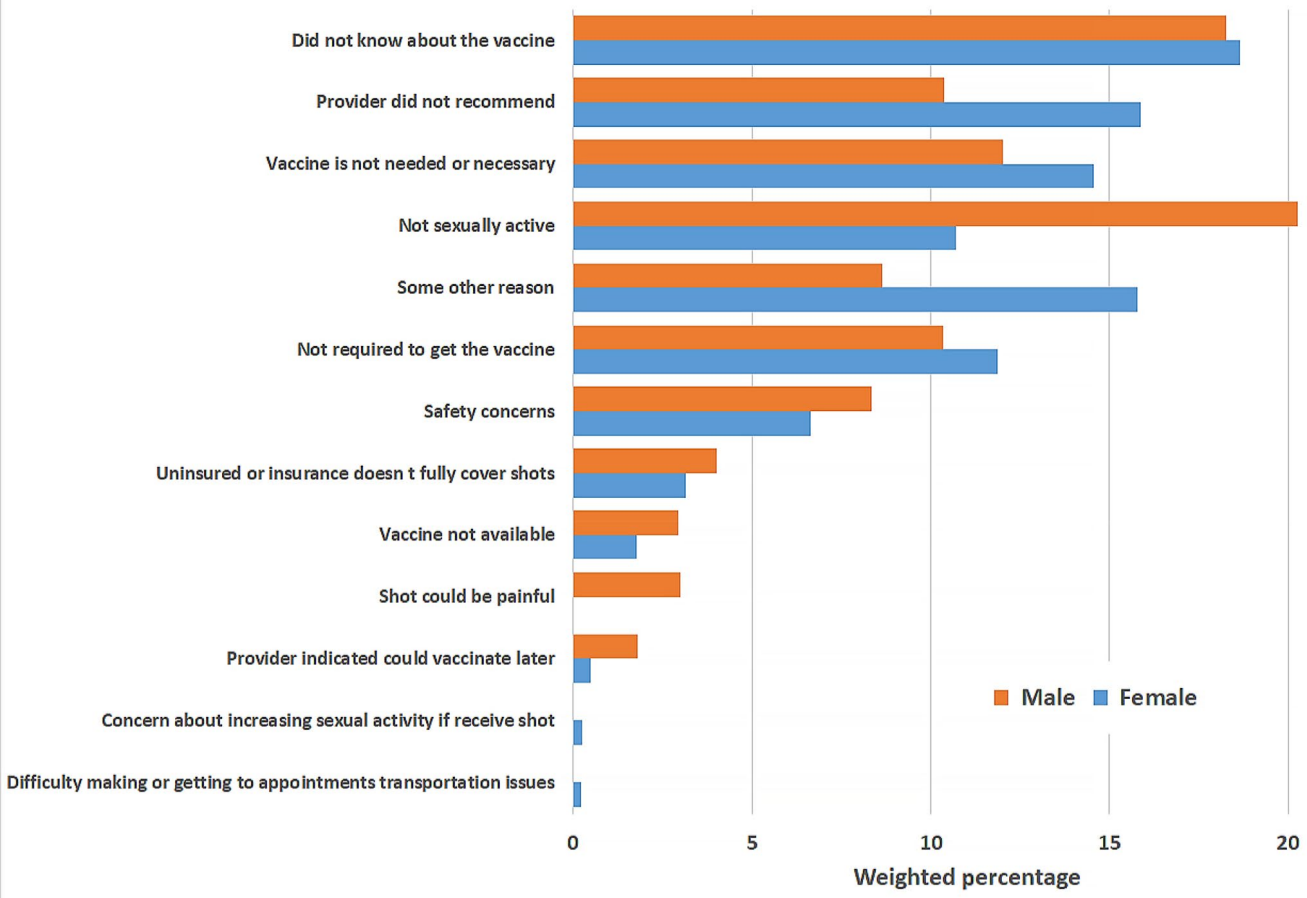
Main Reasons for not getting the HPV vaccine among vaccine‐eligible adults, by sex

**FIGURE 3:**
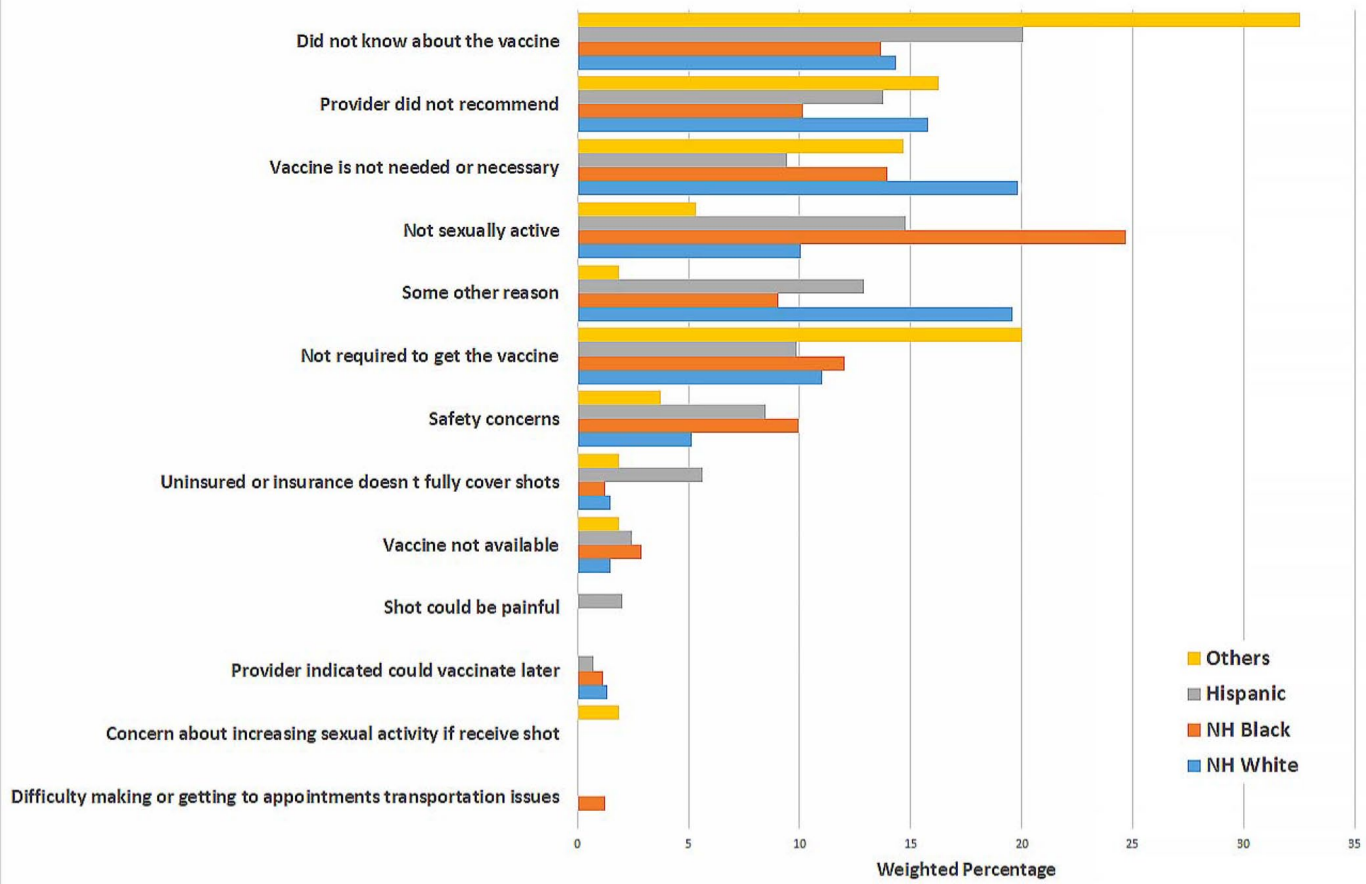
Reasons for not getting the HPV vaccine among vaccine‐eligible adults, by race/ethnicity

**FIGURE 4:**
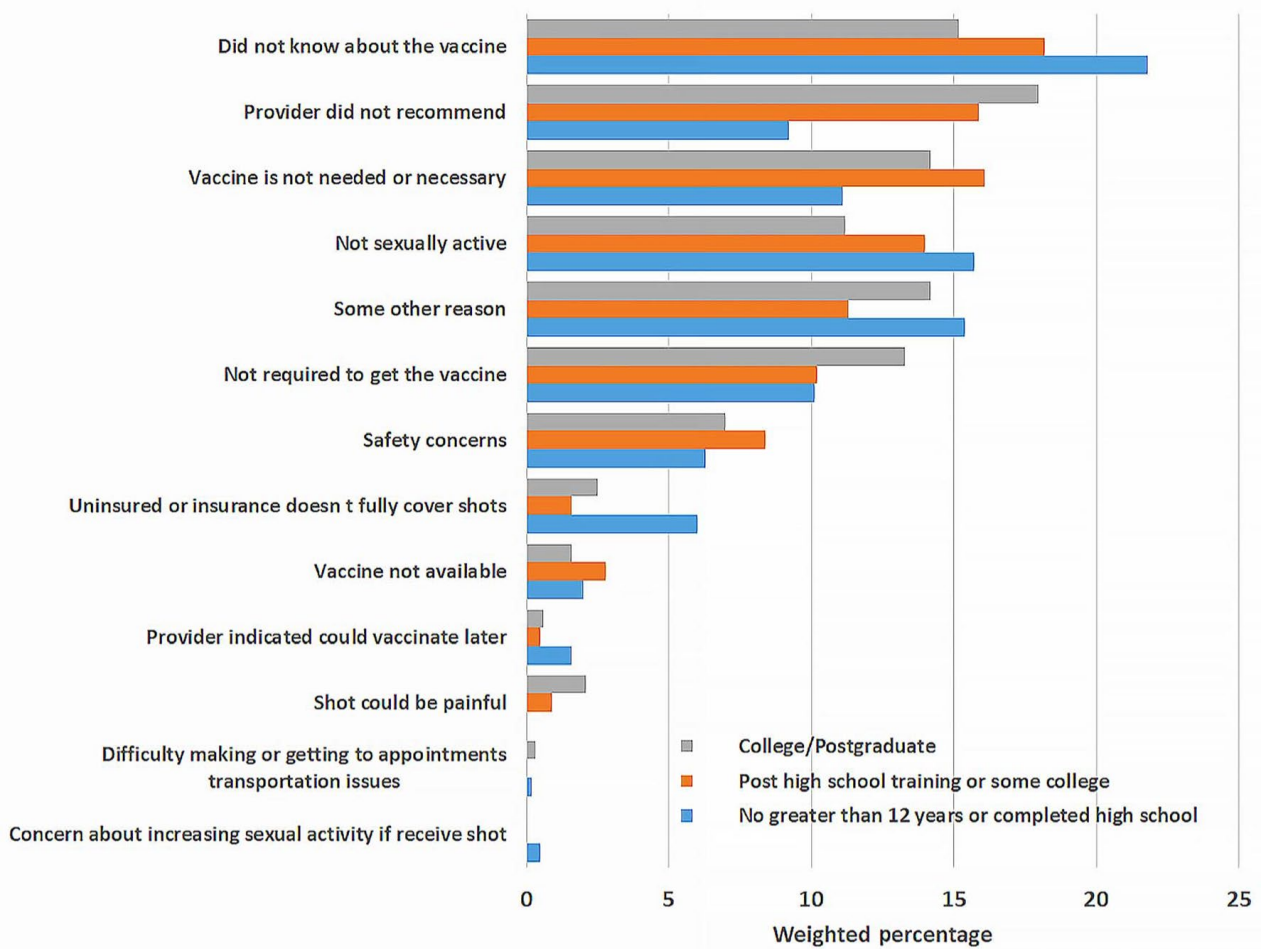
Reasons for not getting the HPV vaccine among vaccine‐eligible adults, by education level

The main reasons for not getting the HPV vaccine differed by certain demographics including sex, ethnicity/race, and education and are reported in Figures 1, 2, 3, 4 and the Table S1. Overall, the most frequently reported reasons were: “did not know about the vaccine” [18.5% (14.9‐22.1)], “provider did not recommend” [14.2% (10.9‐17.4)], “vaccine not needed or necessary” [13.8% (10.5‐17.0)], and “not sexually active” [13.7% (10.5‐16.9)] (Figure 1). Among women, the most common reason reported was ‘did not know about the vaccine’ [18.6% (14.7‐22.6)], while among men, it was “not sexually active” [20.3% (12.7‐27.9)] (Figure 2). Among NHWs, the most common reason was “vaccine not needed or necessary” [19.8% (12.2‐27.5)], while it was “did not know about the vaccine” among Hispanics [20.1% (14.7‐25.4)], and “not sexually active” among NHBs [24.7% (16.8‐32.6)] (Figure 3). Among women with post‐graduate education, the main reason reported was “provider did not recommend” [18.0% (11.3‐24.7)]; while in women with not greater than high school education, the main reason was “did not know about the vaccine” [21.8% (15.3‐28.4)] (Figure 4).

Some of the key least commonly reported reasons for not getting HPV vaccine included: “safety concerns” (7.2% (4.8‐9.5)); “uninsured or insurance doesn't fully cover shots” (3.4% (1.7‐5.1)); “concerns about increasing sexual activity if receive the vaccine” [0.2% (0.0‐0.5)]; and “difficulty making or getting to appointments, or transportation issues” (0.2% (0.0‐0.4)) (Figure 1).

Reasons for not having received HPV vaccination described according to respondents’ characteristics age, origin of birth, marital status, occupational status, income, residency, use of hormonal contraception, smoking status, availability of health care, vaccination status for hepatitis B, and family history of cancer are reported in the Table S1.

## 4 DISCUSSION

In this representative sample of adult Texas residents eligible for HPV vaccination, differences in socio‐demographic, health‐related, and behavioral characteristics were assessed between those who reported having ever received the HPV vaccine, and those who had never been vaccinated for HPV. Among eligible respondents who reported having not received HPV vaccination, we examined the reasons why they did not get the vaccine. To the best of our knowledge, this is the first study to assess individual's reasons for not receiving HPV vaccine in the Texas adult population, a state with an alarmingly low HPV vaccine uptake.

Texas has experienced a 20‐fold increase in nonmedical exemptions of vaccination for students since 2003 when exemptions for religious and personal beliefs were enacted.^15^, ^16^ It is also one of the US States with the lowest HPV vaccination rates.^18^ The growing number of unvaccinated children arriving at Texas schools makes this state increasingly vulnerable to vaccine preventable outbreaks, according to a recent computer simulation.^17^ To help improve HPV vaccination rates, the Texas Medical Association recently joined with more than 40 other organizations to announce a renewed statewide immunization campaign to prevent HPV‐related cancers.^24^ Despite these efforts, the impact that the rising number of anti‐vaccine movements in the State have had on HPV vaccination coverage remains unknown. In order to examine the various reasons expressed by potential adult beneficiaries for the low HPV vaccination uptake, we administered this state‐wide survey. Excluding participants who did not know their HPV vaccination status from our study population, 28.3% of women and 23.8% of men reported having ever received HPV vaccination. Based on the NHIS data, HPV vaccination rates (at least one dose, ever) in 2016 were 48.5% among women aged 19 to 26 years, and 21.2% among men aged 19‐21 years.^5^ Our study finding suggests that the lower HPV vaccination uptake among eligible adults in Texas, as compared to national rates, is driven by relatively lower HPV vaccination rate among adult vaccine‐eligible women in Texas. Further studies are needed to understand barriers to HPV vaccination among women in Texas, and the reasons why they are less likely to be vaccinated for HPV than the average adult woman in the US.

HPV vaccination is routinely administered between 11 and 12 years with parental consent (2). However, catch‐up immunization is recommended for both men and women through early adulthood,^2^ when individuals can independently make an informed choice about their health, including the decision about HPV vaccination. Thus, beyond parents’ perspectives, understanding individuals’ reasons for not receiving HPV vaccine is critical to improving vaccine uptake among eligible adults. In line with the recent FDA approval of HPV vaccine until age 45,^11^ the ACIP has recommended its use among US adults aged 27 to 45 years as part of shared clinical decision‐making.^13^ In this context, it is essential to examine the reasons for non‐initiation of HPV vaccination in the adult eligible population.

In our study sample, lack of knowledge about HPV vaccine, lack of provider's recommendation, and perceived lack of necessity, were the most commonly reported reasons for not getting the vaccine. Interestingly, these reasons varied by certain demographics. Among women, the main reason for not getting the HPV vaccine was “did not know about the vaccine”, while among men, it was “not sexually active.” In a study assessing US parents’ reasons for lack of HPV vaccine initiation in their children, “safety concerns” was most frequently reported by parents of eligible girls vs “lack of necessity” by parents of eligible boys.^25^ It is well known that children's values and practices are generally influenced by their parents’ beliefs and attitudes.^26^, ^27^ Our findings, however, suggest that when they become adults, children may not necessarily give the same reasons as their parents for not receiving HPV vaccine. This highlights the need to develop adapted educational interventions to improve HPV vaccine uptake among eligible adults. The finding that sexual inactivity is a major reason for nonreceipt of HPV vaccine among men, may reflect their poorer understanding of the need to vaccinate before sexual debut. This is consistent with prior literature demonstrating less frequent, weaker, and less timely HPV vaccine recommendations for men compared to women, despite evidence that provider recommendation may have an even stronger impact on men's vaccine initiation.^28^ Because providers who feel that they must discuss sexuality prior to recommending the vaccine tend to deliver lower quality, less frequent recommendations,^28^ targeted interventions are needed to improve the impact of messages that providers deliver to patients. Only 0.2% of respondents (regardless of sex) reported concerns that the vaccine would increase their sexual activity. Therefore, providers’ concerns about discussing sexual activity is not relevant for vaccine‐eligible adult individuals unlike for parents of vaccine‐eligible adolescents,^29^ and thus, should not obstruct delivery of strong recommendations, especially among women.

Although the HPV vaccine was FDA approved to prevent cancer over a decade ago, eligible adults still report a lack of knowledge and understanding of its necessity. However, safety concerns, lack of insurance, transportation issues as well as some other concerns were less common in our study sample, suggesting that reasons commonly highlighted in mainstream media, such as anti‐vaccination beliefs including religious objections are not the prime factors underlying individuals’ perception of HPV vaccination in Texas.^30^

## 5 STRENGTHS AND LIMITATIONS

Strengths of the study include its large sample size and the representativeness of the adult Texas population. The study is somewhat limited, however, in its ability to detect simultaneous reasons individuals may hold, as the question was framed to assess the “main” reason for not getting the vaccine. Because information about whether or how often eligible individuals visited a healthcare provider was not available, we could not distinguish between respondents with missed clinical opportunities for HPV vaccine recommendation, and those unexposed to provider recommendations. While the self‐report of the vaccine status by study participants could yield a certain level of “misclassification” bias, substantial agreement between electronic medical reports of the vaccine status and self‐reported data were noted for HPV vaccination.^31^

## 6 CONCLUSION

In this study, individual reasons for not receiving the HPV vaccine were assessed among eligible adults. A substantial proportion of age‐eligible adults in Texas reported lack of knowledge or lack of provider recommendation as the main reasons for not receiving HPV vaccine. Rather than emphasizing on safety or sexual activity, educational interventions should focus on the necessity and awareness of HPV vaccination. Providers’ recommendation for HPV vaccine should be reinforced, and further adapted to older age groups.

## CONFLICT OF INTEREST

The authors declare no potential conflicts of interest.

## AUTHOR CONTRIBUTIONS

Concept and Study Design: Fokom Domgue, Shete. Data Analysis: Yu. Interpretation of the data: Fokom Domgue, Cunningham, Yu, Shete. Initial Draft: Fokom Domgue. Critical revision of the manuscript for important intellectual content: Fokom Domgue, Cunningham, Yu, Shete. Obtained Funding and Study Supervision: Shete.

## ROLE OF FUNDER/SPONSOR STATEMENT

The funders had no involvement in design and conduct of the study; analysis and interpretation of the data; preparation, review, or approval of the manuscript; and the decision to submit the manuscript for publication.

## Supporting information

TableS1

## Data Availability

The data supporting findings of our study can be obtained from the corresponding author.
