# Supplementary material for: Reasons for not receiving the HPV vaccine among eligible adults: Lack of knowledge and of provider recommendations contribute more than safety and insurance concerns
Source: Cancer Med. 2020 Jun 1;9(14):5281–90. doi: 10.1002/cam4.3192 (PMC7367641; doi:10.1002/cam4.3192)
Supplement: Supplementary file 1 — TableS1 [file CAM4-9-5281-s001.docx]

**Supplemental Table: Self-reported main Reasons for having not received HPV vaccine among eligible Texas adults, according to socio-demographic, health-related and behavioral characteristics**

|  | **Total** | | | | **Did not know about the vaccine** | | | | **Provider did not recommend** | | | | **Vaccine is not needed or necessary** | | | | **Not sexually active** | | | | **Some other reason** | | | | **Not required to get the vaccine** | | | | **Safety concerns** | | | | **Uninsured or insurance doesn't fully cover shots** | | | | **Vaccine not available** | | | | **Shot could be painful** | | | | **Provider indicated could vaccinate later** | | | | **Concern about increasing sexual activity if receive shot** | | | | **Difficulty making or getting to appointments, transportation issues** | | | |
| --- | --- | --- | --- | --- | --- | --- | --- | --- | --- | --- | --- | --- | --- | --- | --- | --- | --- | --- | --- | --- | --- | --- | --- | --- | --- | --- | --- | --- | --- | --- | --- | --- | --- | --- | --- | --- | --- | --- | --- | --- | --- | --- | --- | --- | --- | --- | --- | --- | --- | --- | --- | --- | --- | --- | --- | --- |
| **Variables** | **N** | **%** | **95% CI** | | **N** | **%** | **95% CI** | | **N** | **%** | **95% CI** | | **N** | **%** | **95% CI** | | **N** | **%** | **95% CI** | | **N** | **%** | **95% CI** | | **N** | **%** | **95% CI** | | **N** | **%** | **95% CI** | | **N** | **%** | **95% CI** | | **N** | **%** | **95% CI** | | **N** | **%** | **95% CI** | | **N** | **%** | **95% CI** | | **N** | **%** | **95% CI** | | **N** | **%** | **95% CI** | |
| **Overall** | 530 | 100.0 |  |  | 97 | 18.5 | 14.9 | 22.1 | 75 | 14.2 | 10.9 | 17.4 | 74 | 13.8 | 10.5 | 17.0 | 75 | 13.7 | 10.5 | 16.9 | 70 | 13.5 | 10.4 | 16.7 | 62 | 11.4 | 8.5 | 14.3 | 39 | 7.2 | 4.8 | 9.5 | 17 | 3.4 | 1.7 | 5.1 | 11 | 2.1 | 0.8 | 3.5 | 3 | 0.9 | 0.0 | 2.0 | 4 | 0.9 | 0.0 | 1.9 | 1 | 0.2 | 0.0 | 0.5 | 2 | 0.2 | 0.0 | 0.4 |
| **Sex** |  |  |  |  |  |  |  |  |  |  |  |  |  |  |  |  |  |  |  |  |  |  |  |  |  |  |  |  |  |  |  |  |  |  |  |  |  |  |  |  |  |  |  |  |  |  |  |  |  |  |  |  |  |  |  |  |
| Female | 417 | 68.5 | 63.8 | 73.3 | 77 | 18.6 | 14.7 | 22.6 | 64 | 15.9 | 12.2 | 19.6 | 63 | 14.6 | 11.0 | 18.1 | 49 | 10.7 | 7.6 | 13.8 | 61 | 15.8 | 12.0 | 19.6 | 50 | 11.9 | 8.6 | 15.2 | 28 | 6.7 | 4.1 | 9.2 | 13 | 3.1 | 1.4 | 4.9 | 7 | 1.8 | 0.4 | 3.1 | 0 | . | . | . | 2 | 0.5 | 0.0 | 1.2 | 1 | 0.2 | 0.0 | 0.7 | 2 | 0.2 | 0.0 | 0.6 |
| Male | 113 | 31.5 | 26.7 | 36.2 | 20 | 18.3 | 10.7 | 25.9 | 11 | 10.4 | 4.2 | 16.5 | 11 | 12.0 | 5.1 | 18.9 | 26 | 20.3 | 12.7 | 27.9 | 9 | 8.6 | 3.0 | 14.3 | 12 | 10.3 | 4.6 | 16.1 | 11 | 8.3 | 3.3 | 13.4 | 4 | 4.0 | 0.1 | 7.9 | 4 | 2.9 | 0.0 | 6.0 | 3 | 3.0 | 0.0 | 6.4 | 2 | 1.8 | 0.0 | 4.6 | 0 | . | . | . | 0 | . | . | . |
| **Ethnicity / Race** |  |  |  |  |  |  |  |  |  |  |  |  |  |  |  |  |  |  |  |  |  |  |  |  |  |  |  |  |  |  |  |  |  |  |  |  |  |  |  |  |  |  |  |  |  |  |  |  |  |  |  |  |  |  |  |  |
| Black, non-Hispanic | 133 | 12.9 | 10.6 | 15.3 | 18 | 13.7 | 7.5 | 19.8 | 14 | 10.2 | 4.9 | 15.5 | 21 | 14.0 | 8.2 | 19.7 | 30 | 24.7 | 16.8 | 32.6 | 13 | 9.0 | 4.1 | 13.9 | 17 | 12.0 | 6.4 | 17.6 | 12 | 10.0 | 4.4 | 15.6 | 2 | 1.2 | 0.0 | 2.9 | 3 | 2.9 | 0.0 | 6.2 | 0 | . | . | . | 1 | 1.1 | 0.0 | 3.3 | 0 | . | . | . | 2 | 1.2 | 0.0 | 2.9 |
| Hispanic | 230 | 46.7 | 42.1 | 51.3 | 48 | 20.1 | 14.7 | 25.4 | 35 | 13.8 | 9.4 | 18.1 | 24 | 9.4 | 5.7 | 13.1 | 31 | 14.7 | 9.8 | 19.7 | 31 | 12.9 | 8.5 | 17.3 | 21 | 9.9 | 5.7 | 14.0 | 18 | 8.4 | 4.5 | 12.4 | 12 | 5.6 | 2.4 | 8.9 | 5 | 2.4 | 0.2 | 4.7 | 3 | 2.0 | 0.0 | 4.3 | 2 | 0.7 | 0.0 | 1.7 | 0 | . | . | . | 0 | . | . | . |
| Others | 45 | 8.8 | 6.2 | 11.4 | 14 | 32.5 | 18.0 | 47.0 | 7 | 16.3 | 4.7 | 27.8 | 7 | 14.7 | 4.2 | 25.2 | 2 | 5.3 | 0.0 | 12.8 | 1 | 1.9 | 0.0 | 5.5 | 9 | 20.0 | 7.8 | 32.2 | 2 | 3.7 | 0.0 | 8.9 | 1 | 1.9 | 0.0 | 5.5 | 1 | 1.9 | 0.0 | 5.5 | 0 | . | . | . | 0 | . | . | . | 1 | 1.9 | 0.0 | 5.5 | 0 | . | . | . |
| White, non-Hispanic | 122 | 31.5 | 26.9 | 36.1 | 17 | 14.3 | 7.8 | 20.9 | 19 | 15.8 | 9.0 | 22.6 | 22 | 19.8 | 12.2 | 27.5 | 12 | 10.0 | 4.4 | 15.6 | 25 | 19.6 | 12.5 | 26.7 | 15 | 11.0 | 5.7 | 16.3 | 7 | 5.1 | 1.4 | 8.9 | 2 | 1.5 | 0.0 | 3.5 | 2 | 1.5 | 0.0 | 3.5 | 0 | . | . | . | 1 | 1.4 | 0.0 | 4.0 | 0 | . | . | . | 0 | . | . | . |
| **Education** |  |  |  |  |  |  |  |  |  |  |  |  |  |  |  |  |  |  |  |  |  |  |  |  |  |  |  |  |  |  |  |  |  |  |  |  |  |  |  |  |  |  |  |  |  |  |  |  |  |  |  |  |  |  |  |  |
| No greater than 12 years or completed high school | 181 | 35.1 | 30.6 | 39.5 | 39 | 21.8 | 15.3 | 28.4 | 18 | 9.2 | 4.8 | 13.6 | 19 | 11.1 | 5.7 | 16.5 | 28 | 15.7 | 9.9 | 21.5 | 29 | 15.4 | 9.8 | 20.9 | 18 | 10.1 | 5.5 | 14.7 | 12 | 6.3 | 2.7 | 9.8 | 10 | 6.0 | 2.1 | 10.0 | 4 | 2.0 | 0.0 | 4.0 | 0 | . | . | . | 2 | 1.6 | 0.0 | 4.1 | 1 | 0.5 | 0.0 | 1.4 | 1 | 0.2 | 0.0 | 0.7 |
| Post high school training or some college | 191 | 35.1 | 30.7 | 39.5 | 33 | 18.2 | 12.2 | 24.3 | 30 | 15.9 | 10.3 | 21.5 | 28 | 16.1 | 10.1 | 22.2 | 31 | 14.0 | 8.9 | 19.1 | 21 | 11.3 | 6.4 | 16.1 | 22 | 10.2 | 5.6 | 14.9 | 16 | 8.4 | 4.0 | 12.7 | 3 | 1.6 | 0.0 | 3.5 | 5 | 2.8 | 0.3 | 5.3 | 1 | 0.9 | 0.0 | 2.7 | 1 | 0.5 | 0.0 | 1.4 | 0 | . | . | . | 0 | . | . | . |
| College/Postgraduate | 157 | 29.9 | 25.6 | 34.1 | 25 | 15.2 | 9.2 | 21.1 | 27 | 18.0 | 11.3 | 24.7 | 27 | 14.2 | 8.8 | 19.5 | 16 | 11.2 | 5.5 | 16.9 | 20 | 14.2 | 8.0 | 20.4 | 21 | 13.3 | 7.7 | 18.9 | 11 | 7.0 | 2.7 | 11.3 | 4 | 2.5 | 0.0 | 4.9 | 2 | 1.6 | 0.0 | 3.9 | 2 | 2.1 | 0.0 | 5.0 | 1 | 0.6 | 0.0 | 1.7 | 0 | . | . | . | 1 | 0.3 | 0.0 | 0.8 |
| **Age (years)** |  |  |  |  |  |  |  |  |  |  |  |  |  |  |  |  |  |  |  |  |  |  |  |  |  |  |  |  |  |  |  |  |  |  |  |  |  |  |  |  |  |  |  |  |  |  |  |  |  |  |  |  |  |  |  |  |
| 18-28 | 310 | 63.8 | 59.5 | 68.1 | 60 | 19.7 | 14.9 | 24.6 | 33 | 10.8 | 7.0 | 14.6 | 43 | 14.2 | 9.8 | 18.6 | 60 | 18.2 | 13.6 | 22.8 | 36 | 11.5 | 7.7 | 15.4 | 34 | 11.0 | 7.2 | 14.7 | 21 | 6.5 | 3.6 | 9.4 | 9 | 3.2 | 1.0 | 5.4 | 4 | 1.4 | 0.0 | 2.9 | 3 | 1.5 | 0.0 | 3.1 | 4 | 1.4 | 0.0 | 3.0 | 1 | 0.3 | 0.0 | 0.8 | 2 | 0.2 | 0.0 | 0.6 |
| 29-38 | 220 | 36.2 | 31.9 | 40.5 | 37 | 16.4 | 11.3 | 21.5 | 42 | 20.1 | 14.4 | 25.8 | 31 | 12.9 | 8.3 | 17.5 | 15 | 5.8 | 2.7 | 8.9 | 34 | 17.0 | 11.6 | 22.5 | 28 | 12.1 | 7.6 | 16.7 | 18 | 8.4 | 4.5 | 12.4 | 8 | 3.8 | 1.1 | 6.6 | 7 | 3.4 | 0.8 | 5.9 | 0 | . | . | . | 0 | . | . | . | 0 | . | . | . | 0 | . | . | . |
| **Born In USA** |  |  |  |  |  |  |  |  |  |  |  |  |  |  |  |  |  |  |  |  |  |  |  |  |  |  |  |  |  |  |  |  |  |  |  |  |  |  |  |  |  |  |  |  |  |  |  |  |  |  |  |  |  |  |  |  |
| No | 44 | 7.5 | 5.2 | 9.7 | 12 | 28.4 | 14.0 | 42.8 | 7 | 15.7 | 4.9 | 26.5 | 4 | 7.8 | 0.1 | 15.4 | 5 | 8.7 | 0.9 | 16.4 | 9 | 21.0 | 8.6 | 33.4 | 3 | 5.5 | 0.0 | 11.8 | 2 | 6.5 | 0.0 | 15.5 | 0 | . | . | . | 1 | 4.2 | 0.0 | 12.2 | 0 | . | . | . | 1 | 2.3 | 0.0 | 6.7 | 0 | . | . | . | 0 | . | . | . |
| Yes | 486 | 92.5 | 90.3 | 94.8 | 85 | 17.7 | 14.0 | 21.4 | 68 | 14.0 | 10.7 | 17.4 | 70 | 14.2 | 10.8 | 17.7 | 70 | 14.1 | 10.7 | 17.5 | 61 | 12.9 | 9.7 | 16.2 | 59 | 11.9 | 8.8 | 15.0 | 37 | 7.2 | 4.8 | 9.7 | 17 | 3.7 | 1.9 | 5.5 | 10 | 2.0 | 0.7 | 3.3 | 3 | 1.0 | 0.0 | 2.2 | 3 | 0.8 | 0.0 | 1.8 | 1 | 0.2 | 0.0 | 0.5 | 2 | 0.2 | 0.0 | 0.4 |
| **Marital Status** |  |  |  |  |  |  |  |  |  |  |  |  |  |  |  |  |  |  |  |  |  |  |  |  |  |  |  |  |  |  |  |  |  |  |  |  |  |  |  |  |  |  |  |  |  |  |  |  |  |  |  |  |  |  |  |  |
| Single/Widowed/ Divorced/Separated | 294 | 56.8 | 52.2 | 61.4 | 53 | 18.9 | 13.8 | 23.9 | 35 | 11.1 | 7.3 | 14.9 | 34 | 12.0 | 7.7 | 16.3 | 69 | 22.1 | 17.0 | 27.3 | 31 | 11.0 | 6.9 | 15.1 | 32 | 10.5 | 6.7 | 14.3 | 24 | 7.8 | 4.5 | 11.1 | 5 | 2.2 | 0.2 | 4.1 | 6 | 2.0 | 0.3 | 3.6 | 2 | 1.1 | 0.0 | 2.7 | 2 | 1.1 | 0.0 | 2.7 | 1 | 0.3 | 0.0 | 0.9 | 0 | . | . | . |
| Living as Married/Married | 227 | 43.2 | 38.6 | 47.8 | 43 | 18.2 | 13.1 | 23.4 | 38 | 17.6 | 12.2 | 23.0 | 39 | 16.4 | 11.2 | 21.5 | 6 | 3.3 | 0.4 | 6.2 | 36 | 16.2 | 11.2 | 21.2 | 29 | 12.4 | 8.0 | 16.9 | 14 | 6.3 | 2.9 | 9.6 | 12 | 5.2 | 2.1 | 8.3 | 5 | 2.5 | 0.3 | 4.7 | 1 | 0.7 | 0.0 | 2.2 | 2 | 0.8 | 0.0 | 1.8 | 0 | . | . | . | 2 | 0.4 | 0.0 | 0.9 |
| **Occupation** |  |  |  |  |  |  |  |  |  |  |  |  |  |  |  |  |  |  |  |  |  |  |  |  |  |  |  |  |  |  |  |  |  |  |  |  |  |  |  |  |  |  |  |  |  |  |  |  |  |  |  |  |  |  |  |  |
| Employed | 299 | 57.2 | 52.7 | 61.8 | 57 | 19.1 | 14.3 | 23.9 | 47 | 15.5 | 11.1 | 20.0 | 43 | 13.5 | 9.3 | 17.8 | 30 | 10.3 | 6.4 | 14.2 | 35 | 12.3 | 8.2 | 16.4 | 35 | 10.9 | 7.2 | 14.7 | 27 | 8.8 | 5.4 | 12.3 | 8 | 2.9 | 0.8 | 5.1 | 8 | 3.0 | 0.9 | 5.2 | 3 | 1.7 | 0.0 | 3.5 | 3 | 1.3 | 0.0 | 3.0 | 1 | 0.3 | 0.0 | 0.9 | 2 | 0.3 | 0.0 | 0.7 |
| Homemaker /Unemployed/Disabled | 151 | 28.3 | 24.2 | 32.4 | 26 | 17.4 | 11.0 | 23.9 | 21 | 14.3 | 8.5 | 20.2 | 23 | 15.3 | 9.1 | 21.5 | 23 | 14.1 | 8.2 | 20.0 | 23 | 16.2 | 9.8 | 22.7 | 16 | 10.4 | 5.2 | 15.6 | 6 | 4.0 | 0.7 | 7.2 | 9 | 6.2 | 2.0 | 10.3 | 3 | 1.5 | 0.0 | 3.3 | 0 | . | . | . | 1 | 0.6 | 0.0 | 1.8 | 0 | . | . | . | 0 | . | . | . |
| Student/Retired/Other | 78 | 14.5 | 11.2 | 17.8 | 14 | 18.8 | 9.0 | 28.5 | 7 | 8.8 | 1.6 | 16.0 | 8 | 12.2 | 3.1 | 21.2 | 22 | 27.0 | 16.4 | 37.6 | 11 | 12.8 | 5.1 | 20.6 | 10 | 13.3 | 5.1 | 21.5 | 6 | 7.1 | 1.1 | 13.2 | 0 | . | . | . | 0 | . | . | . | 0 | . | . | . | 0 | . | . | . | 0 | . | . | . | 0 | . | . | . |
| **Income** |  |  |  |  |  |  |  |  |  |  |  |  |  |  |  |  |  |  |  |  |  |  |  |  |  |  |  |  |  |  |  |  |  |  |  |  |  |  |  |  |  |  |  |  |  |  |  |  |  |  |  |  |  |  |  |  |
| ≤ $19,999 | 158 | 28.7 | 24.6 | 32.9 | 22 | 14.0 | 8.2 | 19.9 | 19 | 11.6 | 6.3 | 16.9 | 15 | 11.1 | 5.1 | 17.2 | 36 | 20.0 | 13.5 | 26.6 | 25 | 15.3 | 9.3 | 21.2 | 14 | 9.4 | 4.4 | 14.3 | 11 | 7.1 | 2.6 | 11.7 | 10 | 6.9 | 2.5 | 11.2 | 4 | 2.0 | 0.0 | 4.0 | 1 | 1.1 | 0.0 | 3.2 | 1 | 1.5 | 0.0 | 4.4 | 0 | . | . | . | 0 | . | . | . |
| $20,000 to $49,999 | 169 | 31.3 | 27.0 | 35.5 | 32 | 19.2 | 12.7 | 25.7 | 33 | 19.5 | 13.2 | 25.8 | 23 | 11.0 | 6.3 | 15.7 | 21 | 14.0 | 8.0 | 20.1 | 17 | 9.8 | 5.1 | 14.6 | 22 | 13.1 | 7.5 | 18.7 | 14 | 8.3 | 3.8 | 12.8 | 3 | 1.8 | 0.0 | 4.1 | 2 | 1.7 | 0.0 | 4.2 | 1 | 1.0 | 0.0 | 3.0 | 1 | 0.5 | 0.0 | 1.6 | 0 | . | . | . | 0 | . | . | . |
| $50,000 to $74,999 | 101 | 17.9 | 14.5 | 21.3 | 16 | 17.1 | 9.0 | 25.2 | 11 | 9.9 | 4.0 | 15.7 | 18 | 17.6 | 9.7 | 25.4 | 12 | 12.3 | 5.2 | 19.4 | 14 | 14.7 | 7.2 | 22.2 | 14 | 12.6 | 5.9 | 19.2 | 7 | 6.5 | 1.6 | 11.3 | 2 | 2.7 | 0.0 | 6.5 | 3 | 3.6 | 0.0 | 7.9 | 0 | . | . | . | 2 | 1.8 | 0.0 | 4.2 | 1 | 0.9 | 0.0 | 2.7 | 1 | 0.4 | 0.0 | 1.3 |
| ≥ $75,000 | 102 | 22.1 | 18.1 | 26.2 | 27 | 24.5 | 15.7 | 33.4 | 12 | 13.4 | 5.7 | 21.1 | 18 | 18.0 | 9.7 | 26.3 | 6 | 6.2 | 1.0 | 11.4 | 14 | 15.6 | 7.6 | 23.5 | 12 | 10.7 | 4.5 | 16.9 | 7 | 6.3 | 1.5 | 11.0 | 2 | 1.8 | 0.0 | 4.4 | 2 | 1.7 | 0.0 | 4.1 | 1 | 1.4 | 0.0 | 4.2 | 0 | . | . | . | 0 | . | . | . | 1 | 0.4 | 0.0 | 1.1 |
| **Residence** |  |  |  |  |  |  |  |  |  |  |  |  |  |  |  |  |  |  |  |  |  |  |  |  |  |  |  |  |  |  |  |  |  |  |  |  |  |  |  |  |  |  |  |  |  |  |  |  |  |  |  |  |  |  |  |  |
| Rural | 184 | 39.8 | 35.2 | 44.4 | 26 | 15.4 | 9.6 | 21.2 | 30 | 17.1 | 11.3 | 23.0 | 27 | 15.9 | 10.0 | 21.9 | 25 | 12.4 | 7.4 | 17.4 | 37 | 18.7 | 12.9 | 24.6 | 18 | 9.4 | 5.1 | 13.6 | 9 | 4.7 | 1.5 | 7.9 | 8 | 3.9 | 1.1 | 6.6 | 2 | 1.0 | 0.0 | 2.4 | 0 | . | . | . | 1 | 1.1 | 0.0 | 3.2 | 1 | 0.4 | 0.0 | 1.2 | 0 | . | . | . |
| Urban | 346 | 60.2 | 55.6 | 64.8 | 71 | 20.6 | 16.0 | 25.2 | 45 | 12.2 | 8.6 | 15.8 | 47 | 12.3 | 8.6 | 16.0 | 50 | 14.6 | 10.5 | 18.7 | 33 | 10.1 | 6.5 | 13.6 | 44 | 12.7 | 8.8 | 16.6 | 30 | 8.8 | 5.6 | 12.1 | 9 | 3.1 | 1.0 | 5.3 | 9 | 2.9 | 0.9 | 4.9 | 3 | 1.6 | 0.0 | 3.3 | 3 | 0.8 | 0.0 | 1.7 | 0 | . | . | . | 2 | 0.3 | 0.0 | 0.6 |
| **Hormonal Contraception*** |  |  |  |  |  |  |  |  |  |  |  |  |  |  |  |  |  |  |  |  |  |  |  |  |  |  |  |  |  |  |  |  |  |  |  |  |  |  |  |  |  |  |  |  |  |  |  |  |  |  |  |  |  |  |  |  |
| No | 161 | 25.3 | 21.6 | 29.1 | 33 | 21.9 | 15.1 | 28.7 | 15 | 9.9 | 5.0 | 14.8 | 21 | 13.1 | 7.6 | 18.7 | 35 | 19.4 | 13.1 | 25.7 | 19 | 13.2 | 7.5 | 18.9 | 19 | 11.2 | 6.1 | 16.3 | 9 | 5.6 | 1.9 | 9.3 | 6 | 3.7 | 0.7 | 6.7 | 1 | 0.3 | 0.0 | 0.9 | 0 | . | . | . | 1 | 0.7 | 0.0 | 2.0 | 1 | 0.7 | 0.0 | 1.9 | 1 | 0.3 | 0.0 | 0.9 |
| Yes | 256 | 43.2 | 38.7 | 47.7 | 44 | 16.7 | 12.0 | 21.5 | 49 | 19.4 | 14.3 | 24.5 | 42 | 15.4 | 10.8 | 20.0 | 14 | 5.6 | 2.6 | 8.6 | 42 | 17.3 | 12.3 | 22.3 | 31 | 12.3 | 8.0 | 16.5 | 19 | 7.3 | 3.9 | 10.6 | 7 | 2.8 | 0.7 | 5.0 | 6 | 2.6 | 0.5 | 4.8 | 0 | . | . | . | 1 | 0.4 | 0.0 | 1.2 | 0 | . | . | . | 1 | 0.2 | 0.0 | 0.5 |
| **Smoking** |  |  |  |  |  |  |  |  |  |  |  |  |  |  |  |  |  |  |  |  |  |  |  |  |  |  |  |  |  |  |  |  |  |  |  |  |  |  |  |  |  |  |  |  |  |  |  |  |  |  |  |  |  |  |  |  |
| Current smokers | 111 | 22.7 | 18.7 | 26.7 | 23 | 23.8 | 14.9 | 32.7 | 13 | 8.8 | 3.8 | 13.9 | 12 | 9.5 | 3.6 | 15.4 | 8 | 7.0 | 1.6 | 12.4 | 18 | 17.4 | 9.5 | 25.3 | 19 | 16.2 | 8.9 | 23.4 | 7 | 5.9 | 1.4 | 10.5 | 5 | 4.7 | 0.5 | 8.8 | 3 | 2.1 | 0.0 | 4.7 | 2 | 2.8 | 0.0 | 6.6 | 1 | 1.9 | 0.0 | 5.5 | 0 | . | . | . | 0 | . | . | . |
| Former smokers | 39 | 7.3 | 5.0 | 9.7 | 5 | 12.3 | 1.3 | 23.2 | 6 | 15.6 | 3.9 | 27.3 | 4 | 8.9 | 0.0 | 17.7 | 2 | 5.4 | 0.0 | 13.8 | 9 | 28.2 | 12.5 | 43.8 | 4 | 8.8 | 0.0 | 17.6 | 4 | 9.8 | 0.5 | 19.2 | 3 | 7.7 | 0.0 | 16.3 | 1 | 2.3 | 0.0 | 6.6 | 0 | . | . | . | 0 | . | . | . | 0 | . | . | . | 1 | 1.1 | 0.0 | 3.2 |
| Never smokers | 380 | 70.0 | 65.6 | 74.3 | 69 | 17.5 | 13.4 | 21.5 | 56 | 15.7 | 11.7 | 19.8 | 58 | 15.7 | 11.5 | 19.8 | 65 | 16.8 | 12.7 | 20.9 | 43 | 10.7 | 7.5 | 14.0 | 39 | 10.1 | 6.9 | 13.4 | 28 | 7.3 | 4.5 | 10.2 | 9 | 2.6 | 0.7 | 4.4 | 7 | 2.1 | 0.5 | 3.8 | 1 | 0.5 | 0.0 | 1.3 | 3 | 0.7 | 0.0 | 1.5 | 1 | 0.2 | 0.0 | 0.7 | 1 | 0.1 | 0.0 | 0.3 |
| **Health Care Coverage** |  |  |  |  |  |  |  |  |  |  |  |  |  |  |  |  |  |  |  |  |  |  |  |  |  |  |  |  |  |  |  |  |  |  |  |  |  |  |  |  |  |  |  |  |  |  |  |  |  |  |  |  |  |  |  |  |
| No | 201 | 37.3 | 32.8 | 41.8 | 38 | 19.6 | 13.6 | 25.7 | 20 | 9.1 | 5.1 | 13.2 | 20 | 10.5 | 5.5 | 15.5 | 41 | 19.0 | 13.1 | 24.8 | 30 | 15.6 | 10.2 | 21.1 | 17 | 8.6 | 4.3 | 12.8 | 13 | 5.2 | 2.1 | 8.2 | 13 | 6.9 | 3.0 | 10.8 | 4 | 2.4 | 0.0 | 4.8 | 1 | 0.8 | 0.0 | 2.5 | 3 | 2.1 | 0.0 | 4.6 | 0 | . | . | . | 1 | 0.2 | 0.0 | 0.6 |
| Yes | 329 | 62.7 | 58.2 | 67.2 | 59 | 17.9 | 13.4 | 22.3 | 55 | 17.1 | 12.7 | 21.6 | 54 | 15.7 | 11.4 | 20.0 | 34 | 10.6 | 6.9 | 14.3 | 40 | 12.3 | 8.4 | 16.1 | 45 | 13.1 | 9.2 | 16.9 | 26 | 8.4 | 5.1 | 11.7 | 4 | 1.3 | 0.0 | 2.7 | 7 | 2.0 | 0.4 | 3.6 | 2 | 1.0 | 0.0 | 2.4 | 1 | 0.2 | 0.0 | 0.7 | 1 | 0.3 | 0.0 | 0.8 | 1 | 0.1 | 0.0 | 0.4 |
| **Hepatitis B Virus vaccination** |  |  |  |  |  |  |  |  |  |  |  |  |  |  |  |  |  |  |  |  |  |  |  |  |  |  |  |  |  |  |  |  |  |  |  |  |  |  |  |  |  |  |  |  |  |  |  |  |  |  |  |  |  |  |  |  |
| No | 350 | 66.9 | 62.6 | 71.3 | 59 | 17.1 | 12.8 | 21.4 | 42 | 12.2 | 8.4 | 15.9 | 49 | 14.1 | 10.0 | 18.1 | 64 | 17.7 | 13.3 | 22.1 | 51 | 14.5 | 10.6 | 18.5 | 35 | 9.9 | 6.5 | 13.2 | 25 | 6.9 | 4.1 | 9.7 | 12 | 3.6 | 1.5 | 5.8 | 5 | 1.5 | 0.1 | 2.9 | 2 | 0.9 | 0.0 | 2.2 | 3 | 1.1 | 0.0 | 2.5 | 1 | 0.2 | 0.0 | 0.7 | 2 | 0.2 | 0.0 | 0.6 |
| Yes | 180 | 33.1 | 28.7 | 37.4 | 38 | 21.4 | 14.9 | 27.8 | 33 | 18.2 | 12.1 | 24.2 | 25 | 13.1 | 7.7 | 18.6 | 11 | 5.7 | 2.2 | 9.2 | 19 | 11.5 | 6.3 | 16.7 | 27 | 14.5 | 8.9 | 20.1 | 14 | 7.7 | 3.4 | 12.0 | 5 | 3.0 | 0.3 | 5.7 | 6 | 3.4 | 0.5 | 6.3 | 1 | 1.0 | 0.0 | 2.8 | 1 | 0.5 | 0.0 | 1.5 | 0 | . | . | . | 0 | . | . | . |
| **Family History of any cancer** |  |  |  |  |  |  |  |  |  |  |  |  |  |  |  |  |  |  |  |  |  |  |  |  |  |  |  |  |  |  |  |  |  |  |  |  |  |  |  |  |  |  |  |  |  |  |  |  |  |  |  |  |  |  |  |  |
| No | 206 | 36.5 | 32.1 | 41.0 | 45 | 22.0 | 15.7 | 28.2 | 26 | 13.3 | 7.9 | 18.7 | 29 | 13.8 | 8.4 | 19.1 | 32 | 14.6 | 9.3 | 19.8 | 20 | 10.0 | 5.5 | 14.5 | 19 | 9.3 | 5.0 | 13.5 | 16 | 7.9 | 3.9 | 11.8 | 7 | 3.2 | 0.8 | 5.6 | 6 | 3.4 | 0.5 | 6.4 | 1 | 0.9 | 0.0 | 2.6 | 3 | 1.3 | 0.0 | 2.9 | 0 | . | . | . | 2 | 0.4 | 0.0 | 1.0 |
| Not sure | 57 | 12.7 | 9.4 | 16.0 | 12 | 20.7 | 9.5 | 31.8 | 5 | 8.4 | 1.0 | 15.7 | 7 | 10.4 | 2.0 | 18.9 | 13 | 25.2 | 12.7 | 37.7 | 12 | 19.6 | 8.8 | 30.3 | 3 | 4.5 | 0.0 | 9.6 | 3 | 6.3 | 0.0 | 13.5 | 1 | 2.5 | 0.0 | 7.3 | 0 | . | . | . | 1 | 2.5 | 0.0 | 7.3 | 0 | . | . | . | 0 | . | . | . | 0 | . | . | . |
| Yes | 266 | 50.8 | 46.1 | 55.4 | 40 | 15.6 | 10.9 | 20.3 | 44 | 16.3 | 11.6 | 20.9 | 38 | 14.6 | 9.9 | 19.3 | 29 | 10.0 | 6.2 | 13.7 | 38 | 14.6 | 10.0 | 19.2 | 40 | 14.7 | 10.1 | 19.3 | 20 | 6.9 | 3.8 | 10.1 | 9 | 3.8 | 1.2 | 6.4 | 5 | 1.8 | 0.2 | 3.3 | 1 | 0.6 | 0.0 | 1.8 | 1 | 0.8 | 0.0 | 2.5 | 1 | 0.3 | 0.0 | 1.0 | 0 | . | . | . |

*** *Male participants did not respond to the question about hormonal contraception use*

*.=sample size too small to yield estimates of percentages and confidence intervals*
